# The Clinical Effect of Annonaceae Fruit Consumption on Caribbean Parkinson′s Disease Severity

**DOI:** 10.1155/bn/8897550

**Published:** 2026-05-26

**Authors:** Louis Ribeyron, Aimée Petit, Régine Edragas, Sandrine Belson, Benoît Tressières, Anne-Catherine Bachoud-Lévi, Philippe Remy, Emmanuel Roze, Laurent Cleret de Langavant, Annie Lannuzel

**Affiliations:** ^1^ Hôpital Universitaire Henri Mondor, Centre Expert Parkinson, Service de Neurologie, AP-HP, Créteil, France, aphp.fr; ^2^ INSERM U955, Institut Mondor de Recherche Biomédicale, Equipe NeuroPsychologie Interventionnelle, Université Paris Est Créteil, Créteil, France, inserm.fr; ^3^ Département d′Etudes Cognitives, Ecole normale supérieure, PSL University, Paris, France, ens.fr; ^4^ Service de Médecine Physique et Réadaptation, Centre Hospitalier Universitaire de Martinique, Fort-de-France, France; ^5^ Service de Neurologie, Centre Hospitalier Universitaire de Guadeloupe, Pointe-à-Pitre/Abymes, France; ^6^ Caribbean Clinical Investigation Center, Inserm CIC 2504, University Hospital of Guadeloupe, Pointe-à-Pitre, France; ^7^ Hôpital de la Pitié-Salpêtrière, DMU Neurosciences, AP-HP, Paris, France, aphp.fr; ^8^ Institut National de la Santé et de la Recherche Médicale, Centre National de la Recherche Scientifique, Institut du Cerveau, Faculté de Médecine de Sorbonne Université, Paris, France, inserm.fr; ^9^ Faculté de Médecine de l′Université des Antilles, Pointe-à-Pitre, France

**Keywords:** acetogenin, Annonaceae fruits, cognition, Parkinson′s disease, risk factor

## Abstract

Annonaceae fruits such as soursop (*Annona muricata*) may worsen symptoms of Parkinson′s disease (PD) in tropical regions. Here, we investigate whether PD is more severe in an Annonaceae‐exposed compared with a nonexposed population. Motor and cognitive symptoms of two PD groups (Caribbean, *N* = 74; mainland France, *N* = 104) were compared after imputation, propensity matching, and multivariate adjustment according to age, disease duration, education, and dopaminergic therapy. Ninety‐six percent of Caribbean (71/74) but none of interviewed mainland France (0/20) PD patients had been exposed to Annonaceae fruits. Caribbean PD patients exhibited more severe motor and cognitive symptoms than mainland France PD patients (Unified PD Rating Scale − 3 = 24.39 [± 14.06] vs. 18.50 [± 11.13], *p* < 0.001; Mattis Dementia Rating Scale = 127.21 [±14.30] vs. 130.80 [± 11.15], *p* = 0.005). Given the growing evidence supporting Annonaceae fruits toxicity, health policy makers should raise public awareness about the risks of such consumption.

**Trial Registration:**

ClinicalTrials.gov identifier: NCT03368300

## 1. Introduction

Annonaceae fruits such as soursop (*Annona muricata)* contain neurotoxic components such as acetogenins which induce mitochondrial dysfunction and tau‐related neurodegeneration in both dopaminergic and nondopaminergic neurons in animal models [1–3]. These fruits are common and consumed for their taste and for traditional medicine applications in tropical areas, including the Caribbean region.

For two decades, the consumption of Annonaceae fruits has been associated with the high proportion of atypical parkinsonism in the Caribbean region [1, 4, 5]. Until recently, idiopathic Parkinson′s disease (PD) patients were thought to be rarely exposed to these neurotoxic fruits. However, in a group of 180 Caribbean patients [6] with either atypical parkinsonism (*N* = 116) or idiopathic PD (*N* = 74), more than 95% of both groups had been exposed. A dose–response relationship was observed between exposure to Annonaceae and worsening of both cognitive and motor symptoms in both groups [7]. PD might be more severe in the Caribbean region compared with areas without exposition to Annonaceae fruits.

To confirm this hypothesis, we compare the clinical phenotype of two groups of PD patients, one from the Caribbean region and one from mainland France.

## 2. Material and Methods

### 2.1. Participants

The Caribbean group consisted of 74 patients with PD from a prospective study of degenerative parkinsonism in French Caribbean islands (Guadeloupe and Martinique) [6, 7]. Patients were examined between 2012 and 2016 and fulfilled the UK PD Society Brain Bank criteria for PD for idiopathic PD. [8]

The mainland France group consisted of 104 PD patients retrospectively and randomly selected from the clinical cohort followed up at the PD expert center in the neurology department at Henri Mondor Hospital in Créteil between 2005 and 2016. We included patients aged over 40, born in mainland France, with idiopathic PD [8], and available motor and cognitive assessment within a 6‐month duration period. We excluded patients with atypical parkinsonism, a history of residence in tropical areas, deep brain stimulation (because it would affect group matching), and associated neurological diseases such as stroke.

### 2.2. Annonaceae Exposure

In the Caribbean, all patients completed a questionnaire about their past consumption of Annonaceae products, allowing for a quantitative estimation of individual exposure to fruits, juices, and herbal teas, expressed in fruit‐or‐cup‐year unit [6, 7]. In mainland France, many patients had passed away by the time of data analysis or were unable to answer the consumption questionnaire. Therefore, we only interviewed 20 of them (17%) about their past consumption of Annonaceae fruits. Note that, unlike in the Caribbean where these fruits are commonly found in private gardens and fresh markets, Annonaceae fruits are rare in mainland France and are only sold in specialized exotic markets.

### 2.3. Clinical Assessment

Both PD groups underwent similar assessments in the ON state, under their usual treatment. We collected the following data: age, sex, disease duration since onset, education according to ISCED 1997, levodopa equivalent daily dose (LEDD) [9], and UPDRS‐3 motor evaluation. Global cognitive efficiency was assessed with the Mattis Dementia Rating Scale (MDRS) [10], attention and working memory with forward and backward digital spans, processing speed with the time for completing the Trail Making Test A (TMTA), executive functions with Frontal Assessment Battery (FAB) [11], verbal episodic memory with the Free and Cued Selective Reminding Test (FCSRT) [12], and language with the 80‐item naming test (DO80) [13].

### 2.4. Imputation and Matching

Missing values were imputed separately in each group of patients with missMDA package [14]. The two groups were then matched according to age, education, disease duration, and LEDD using optimal full matching with the MatchIt package [15]. Balance between groups after matching was assessed using standardized mean differences (SMDs), empirical cumulative distribution functions (eCDFs), and propensity scores.

### 2.5. Statistical Analysis

Differences in motor and cognitive severity between matched groups were tested using multivariate linear regression models, adjusted for age, disease duration, education, and LEDD, and weighted using optimal full matching.

## 3. Results

### 3.1. Group Comparison Before Matching

Caribbean and mainland France PD groups had similar age (*p* = 0.755) and sex ratio (*p* = 0.516) (Table 1). However, the Caribbean PD group had lower education levels (*p* = 0.008), shorter disease duration (*p* = 0.007), and received lower doses of dopaminergic therapy (*p* < 0.001) compared with the mainland France PD group.

**Table 1:** Demographic and clinical characteristics of the two PD groups, mean (min–max; standard deviation). Abbreviations: *N*: number of observations (Mainland/Caribbean); *p*: result of the *p* value for unadjusted comparison between groups; *p*_adjusted_: result of the *p* value of group comparison after matching and multivariate analysis adjusted with the remaining confounding factors as appropriate (age, sex, education, disease duration, and LEDD); FAB: Frontal Assessment Battery; FCSRT: Free and Cued Selective Reminding Test; DO80: denomination 80 items; TMTA: Trail Making Test A; LEDD: levodopa equivalent daily dose; Mattis DRS: Mattis Dementia Rating Scale; UPDRS‐3: Unified Parkinson′s Disease Rating Scale 3.

	*N*	Mainland France	Caribbean region	*p*	*p*_adjusted_
Demographics					
Age (years)	104/74	67.0 (44–84; 9.07)	67.41 (47–81; 8.09)	0.755	0.914
Sex (M/F)	104/74	68/36	44/30	0.516	0.311
Disease duration (years)	104/74	9.45 (0–27; 5.62)	7.19 (0–23; 5.36)	0.007	0.265
Education above secondary school	104/74	73.1%	52.7%	0.008	0.621
LEDD (mg)	68/74	1046 (150–2521; 533)	596 (0–2250; 397)	< 0.001	0.247
Motor score					
UPDRS 3	104/74	18.50 (1–57; 11.13)	24.39 (4–72; 14.06)	0.003	< 0.001
Cognitive assessment					
Mattis DRS	91/70	130.80 (91–144; 11.15)	127.21 (84–144; 14.30)	0.086	0.005
TMTA	42/62	97.43 (30–414; 75.17)	98.13 (25–331; 61.36)	0.960	0.051
Forward digital span	101/69	5.46 (3–9; 1.22)	4.97 (0–9; 1.31)	0.016	0.001
Backward digital span	102/68	3.65 (2–8; 1.15)	3.38 (0–7; 1.13)	0.140	0.250
FAB	75/69	13.68 (3–18; 3.25)	12.46 (4–18; 3.42)	0.031	0.014
FCSRT immediate recall	95/43	14.67 (8–16; 1.66)	15.07 (9–16; 1.55)	0.177	0.467
FCSRT sum free recalls	95/42	22.87 (1–39; 8.05)	23.81 (8–37; 7.61)	0.516	0.046
FCSRT sum total recalls	95/42	44.12 (26–48; 4.16)	41.76 (24–48; 6.18)	0.028	< 0.001
FCSRT recognition	95/43	15.67 (13–16; 0.63)	15.77 (14–16; 0.53)	0.349	0.142
FCSRT delayed free recall	95/42	9.17 (0–16; 3.63)	8.17 (0–16; 3.93)	0.163	< 0.001
FCSRT delayed total recall	95/41	14.88 (7–16; 1.75)	14.29 (7–16; 2.91)	0.235	< 0.001
DO80	85/68	77.11 (28–80; 5.92)	72.13 (53–80; 6.58)	< 0.001	< 0.001

Ninety‐six percent (71/74) of Caribbean PD patients had consumed Annonaceae products. Their mean Annonaceae fruit consumption reached 21.8 fruit‐or‐cup units (SD 47.6), equivalent to consuming everyday either one Annonaceae fruit or one glass of Annonaceae juice or one cup of Annonaceae leaves infusion for 21.8 years. None of the 20 interviewed PD patients from mainland France had been exposed.

### 3.2. Assessing Balance After Matching

The matching method limited the effects of differences between groups in terms of education, disease duration, and dopaminergic therapy. Improved balance is suggested by the examination of eCDFs plots, SMDs, and propensity score (Figure S1). The SMD of the distance between matched groups is 0.025. LEDD differences between groups were balanced after matching (absolute SMD = 0.049).

### 3.3. Group Differences

**Figure 1:**
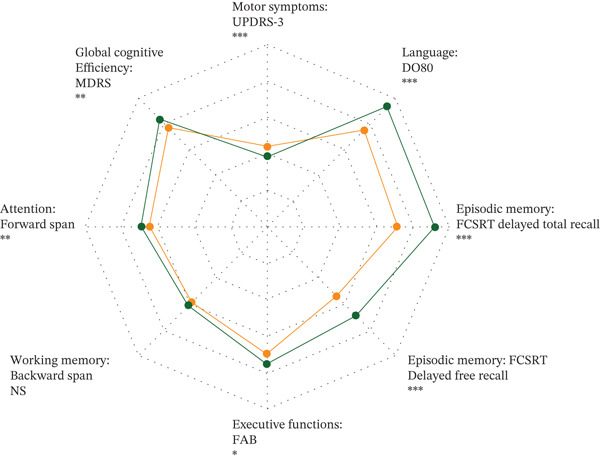
Clinical differences between Caribbean and mainland France PD patients. For each group, we show predicted means of motor and cognitive measures according to multivariate models adjusted for age, education, disease duration, and dopaminergic treatment (see Table S1). The spider plot is scaled according to the range of observed data in the study. Symbols denote the range of the *p* value: *p* < 0.001 ( ^∗∗∗^), *p* < 0.01 ( ^∗∗^), *p* < 0.05 ( ^∗^), *p* > 0.05 (NS). Detailed results are reported in Table S1.

After matching and adjustment for age, education, disease duration and LEDD in multivariate analyses, the Caribbean PD group not only exhibited more severe motor symptoms than the mainland France group (*p* < 0.001), but also showed lower performance at tests assessing global cognitive efficiency, attention, executive functions, verbal episodic memory and language (all *p* < 0.05; Figure 1 and Table 1).

### 3.4. Sensitivity Analysis

We analyzed a smaller sample composed of the Caribbean group (*N* = 74) and the mainland group with all LEDD available (*N* = 68) without using imputation. We used a matching procedure based on age, education, disease duration, and LEDD. Multivariate analyses showed more severe motor symptoms and lower cognitive performance at tests assessing attention, verbal episodic memory, and language in the Caribbean group (Table S1).

We also tested group differences after excluding the three patients from the Caribbean group that were not exposed to Annonaceae fruits and found similar results as in Table 1 (results not shown).

### 3.5. Post Hoc Analyses

Consumption of Annonaceae above a 2 fruit‐or‐cup‐year threshold (see Methods) in the Caribbean group was associated with a mean 8.3 increase (95% CI: 1.2–15; *p* = 0.026) in the UPDRS‐3 motor score as determined by a multivariate regression analysis adjusted for age, sex, education, disease duration, and comorbidities (hypertension, diabetes, dyslipidemia, and obesity). Using similar multivariate analyses, consumption of Annonaceae products above a 0.3 fruit‐or‐cup‐year threshold in the Caribbean group was associated with a mean 5.7 decrease (95% CI: 0.39–11; *p* = 0.04) in linguistic performance on the DO80 test.

## 4. Discussion

The phenotype of PD is more severe regarding motor and cognitive symptoms in the Caribbean region compared with mainland France. In contrast to patients from mainland France, Caribbean PD patients are very often exposed to Annonaceae fruits. A dose–effect relationship is found between Annonaceae fruits consumption and both motor and cognitive performance in Caribbean PD patients.

Despite some limitations, we speculate that consumption of Annonaceae fruits accounts for the more severe phenotype of PD in the Caribbean region. We acknowledge that data from the Caribbean were collected prospectively and data from mainland France retrospectively. Caribbean patients with very low education, severe cognitive impairment, or dysarthria were not proposed a neuropsychological assessment [6, 7]. Conversely, PD patients in mainland France benefited from a cognitive assessment in case of cognitive complaints, suspicion of cognitive impairment, or consideration for subthalamic stimulation surgery. This may have yielded the recruitment of patients with more severe cognitive or motor impairment in mainland France. Yet, these recruitment biases might have diminished clinical differences between the groups. Moreover, consumption of Annonaceae fruits was only evaluated in a small subset of mainland France patients, with the assumption that the whole group was not exposed. However, we insist that Annonaceae fruits are difficult to find in mainland France unlike in the Caribbean region. Again, if some PD patients in mainland France had consumed Annonaceae fruits, this would have likely reduced the clinical differences between the groups. The two groups also have different genetic backgrounds and ancestries, which were not assessed in this study. Additionally, comorbidities such as hypertension, diabetes, smoking, alcohol consumption, or sleep disorders were not explored in the mainland France group. However, post hoc analyses that considered comorbidities confirm a dose–effect relationship between Annonaceae consumption and clinical severity in the Caribbean group.

Language and verbal episodic memory are more affected in Caribbean patients. Although group differences in education were considered in the analyses, variations in French language proficiency may affect cognitive performance. Indeed, Caribbean patients often speak both Creole and French languages. However, only Caribbean patients with adequate French language proficiency were offered a neuropsychological assessment. Additionally, the forward digit span is likely less influenced by a potential linguistic bias and still differs between groups. Lastly, the negative association between Annonaceae consumption and linguistic performance in the Caribbean group in post‐hoc analyses validates the cognitive impact of fruit exposure.

The group differences in LEDD before matching can be attributed to varying clinical practices across centers or lower Dopa‐sensitivity in the Caribbean group [5, 6]. However, LEDD differences probably cannot explain group differences regarding motor function because of the matching and multivariate analysis procedure. Furthermore, the longer disease duration observed in the mainland France group could have yielded more severe motor and cognitive symptoms in this group, which was not the case.

Both the motor and cognitive phenotypes of PD are more severe in the French Caribbean region compared with mainland France, possibly due to exposure to Annonaceae fruits. Annonaceae fruits should be considered a potential threat to a larger population than previously believed, as they may cause both cognitive and motor impairment in PD.

## Author Contributions

Conceptualization: L.C.L. and A.L.; data curation: A.P., R.E., S.B., and L.R.; formal analysis: L.R., L.C.L., and B.T.; supervision: L.C.L. and A.L.; writing—original draft: L.R. and A.P.; writing—review and editing: all remaining authors. L.C.L. and A.L. contributed to the work equally and should be regarded as co‐first authors.

## Funding

This work was funded by the French Ministry of Health (Interregional Hospital Clinical Research Program 2011‐A01259‐32), Guadeloupe Region and the European Union Through REG‐MND (Registre Guadeloupéen des Maladies Neurodégénératives; 2019‐FED‐118), the French National Research Agency (ANR‐17‐EURE‐0017 grant), and France Parkinson Association.

## Disclosure

The authors have nothing to report.

## Ethics Statement

The Caribbean CP study was approved by the French Sud Ouest and Outre Mer III Ethics Committee (2011/86). Written informed consent was obtained from all patients in the CP study. The Henri Mondor Institutional Review Board (IRB) approved the retrospective data analysis for patients in the mainland cohort (N°2024‐185). Following the recommendations of Henri Mondor IRB, the written informed consent was not necessary for the retrospective analysis of patients in the mainland cohort. Many mainland patients had already passed away at the time of the analysis. The remaining patients received an information letter giving them the opportunity to opt out for their participation in the study. One month after having received the information letter, their data could be analyzed according to the IRB. We confirm that we have read the journal′s position on issues involved in ethical publication and affirm that this work is consistent with those guidelines.

## Conflicts of Interest

The authors declare no conflicts of interest.

## Supporting Information

Additional supporting information can be found online in the Supporting Information section.

## Supporting information


**Supporting Information 1** Table S1: Group comparisons after matching and adjustment in multivariate analyses without imputation. Abbreviations: FAB: Frontal Assessment Battery; FCSRT: Free and Cued Selective Reminding Test; DO80: denomination 80 items; TMTA: Trail Making Test A; LEDD: levodopa equivalent daily dose; Mattis DRS: Mattis Dementia Rating Scale; UPDRS‐3: Unified Parkinson′s Disease Rating Scale 3.

**Supporting Information 2** Figure S1: Assessing balance between matched groups. (A) Empirical cumulative distribution functions (eCDFs) plots, in the whole sample and in matched sample. Black curves correspond to the Caribbean group, grey ones to mainland France. Matched curves are better aligned than unmatched ones. (B) Absolute standardized mean differences (SMDs) before (black) and after (grey) matching. Values closer to zero denote balanced groups. (C) Propensity scores before (left panels) and after (right panels) matching, in both groups (upper histogram: mainland France, bottom: Caribbean region).

## Data Availability

The data that support the findings of this study are available from the corresponding author upon reasonable request.
