# Supplementary material for: The Clinical Effect of Annonaceae Fruit Consumption on Caribbean Parkinson′s Disease Severity
Source: Behav Neurol. 2026 May 26;2026:8897550. doi: 10.1155/bn/8897550 (PMC13212262; doi:10.1155/bn/8897550)
Supplement: Supplementary file 1 — Supporting Information 1 Table S1: Group comparisons after matching and adjustment in multivariate analyses without imputation. Abbreviations: FAB: Frontal Assessment Battery; FCSRT: Free and Cued Selective Reminding Test; DO80: denomination 80 items; TMTA: Trail Making Test A; LEDD: levodopa equivalent daily dose; Mattis DRS: Mattis Dementia Rating Scale; UPDRS‐3: Unified Parkinson′s Disease Rating Scale 3. [file BN-2026-8897550-s001.docx]

|  | N | beta | P-value |
| --- | --- | --- | --- |
| Motor assessment | | | |
| UPDRS motor score | 142 | 5.16 | **0.012** |
| Cognitive assessment | | | |
| Mattis DRS | 130 | -3.68 | 0.090 |
| Forward digital span | 137 | -0.53 | **0.012** |
| Backward digital span | 136 | -0.05 | 0.776 |
| TMTA | 90 | 20.49 | 0.094 |
| FAB | 119 | -0.59 | 0.278 |
| FCSRT immediate recall | 106 | 0.30 | 0.280 |
| FCSRT sum free recalls | 104 | 2.14 | 0.148 |
| FCSRT sum total recalls | 104 | -2.80 | **0.003** |
| FCSRT recognition | 105 | -0.06 | 0.506 |
| FCSRT delayed free recall | 102 | -0.99 | 0.183 |
| FCSRT delayed total recall | 102 | -1.05 | **0.010** |
| DO80 | 124 | -4.99 | **<0.001** |
