# Supplementary material for: The Clinical Effect of Annonaceae Fruit Consumption on Caribbean Parkinson′s Disease Severity
Source: Behav Neurol. 2026 May 26;2026:8897550. doi: 10.1155/bn/8897550 (PMC13212262; doi:10.1155/bn/8897550)
Supplement: Supplementary file 2 — Supporting Information 2 Figure S1: Assessing balance between matched groups. (A) Empirical cumulative distribution functions (eCDFs) plots, in the whole sample and in matched sample. Black curves correspond to the Caribbean group, grey ones to mainland France. Matched curves are better aligned than unmatched ones. (B) Absolute standardized mean differences (SMDs) before (black) and after (grey) matching. Values closer to zero denote balanced groups. (C) Propensity scores before (left panels) and after (right panels) matching, in both groups (upper histogram: mainland France, bottom: Caribbean region). [file BN-2026-8897550-s002.pptx]

## Slide 1
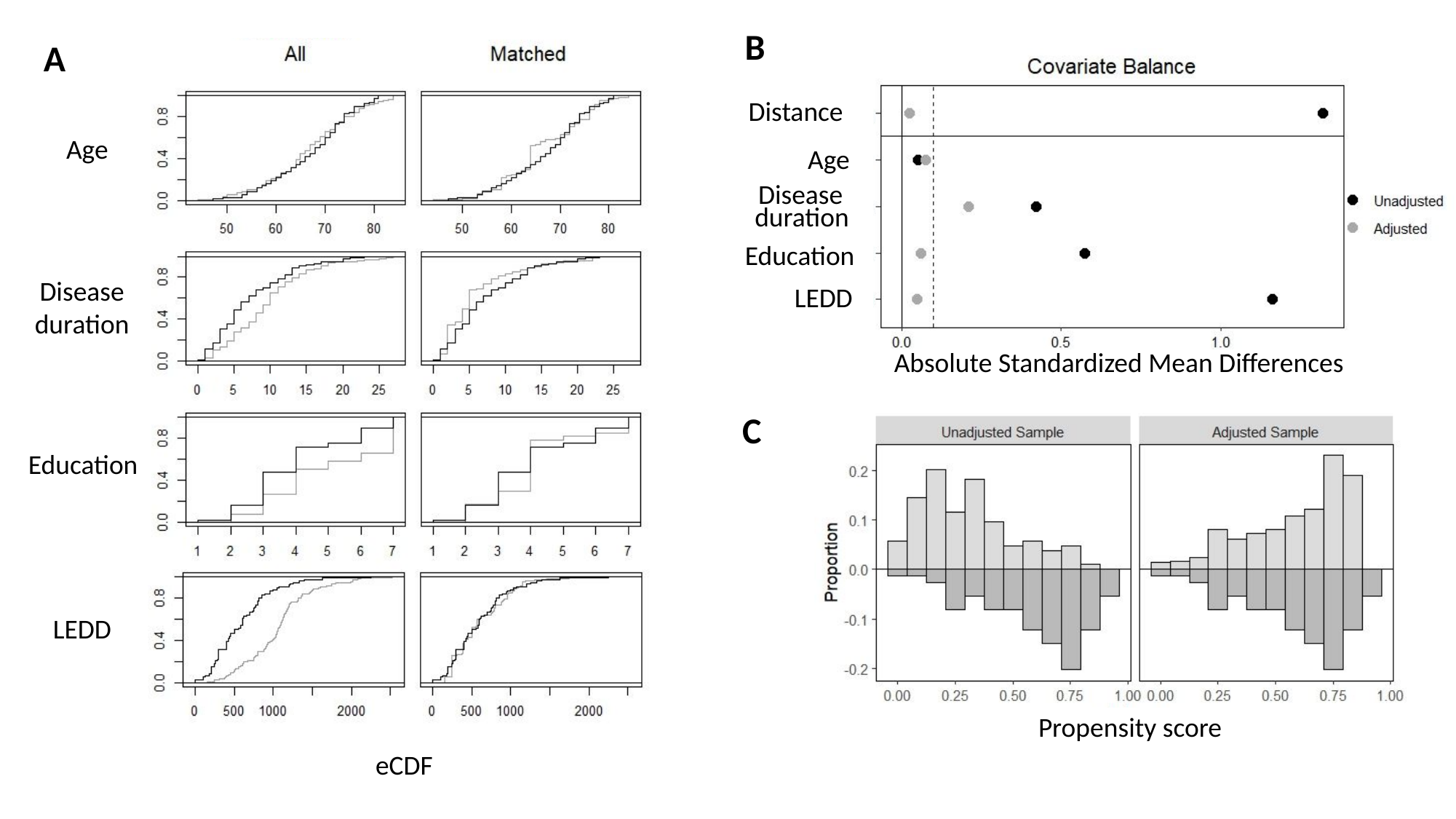

B
A
Distance
Age
Age
Disease
duration
Education
Disease duration
LEDD
Absolute Standardized Mean Differences
C
Education
LEDD
Propensity score
eCDF
